# Risk Factors and Prediction of Stroke in a Population with High Prevalence of Diabetes: The Strong Heart Study

**DOI:** 10.4236/wjcd.2017.75014

**Published:** 2017-05-27

**Authors:** Wenyu Wang, Ying Zhang, Elisa T. Lee, Barbara V. Howard, Richard B. Devereux, Shelley A. Cole, Lyle G. Best, Thomas K. Welty, Everett Rhoades, Jeunliang Yeh, Tauqeer Ali, Jorge R. Kizer, Hooman Kamel, Nawar Shara, David O. Wiebers, Julie A. Stoner

**Affiliations:** 1 College of Public Health, University of Oklahoma Health Sciences Center, Oklahoma City, OK, USA; 2 MedStar Health Research Institute, Hyattsville, MD, USA; 3 Weill Cornell Medical College, New York, NY, USA; 4 Texas Biomedical Research Institute, San Antonio, TX, USA; 5 Missouri Breaks Industries Research Inc., Eagle Butte, SD, USA; 6 Aberdeen Area Tribal Chairmen’s Health Board, Rapid City, SD, USA; 7 College of Medicine, University of Oklahoma Health Sciences Center, Oklahoma City, OK, USA; 8 Albert Einstein College of Medicine, Bronx, NY, USA; 9 Mayo Clinic and Mayo Foundation, Rochester, MN, USA

**Keywords:** Stroke, Risk Factors, Prediction, Prevention

## Abstract

**Background and Objective:**

American Indians have a high prevalence of diabetes and higher incidence of stroke than that of whites and blacks in the U.S. Stroke risk prediction models based on data from American Indians would be of clinical and public health value.

**Methods and Results:**

A total of 3483 (2043 women) Strong Heart Study participants free of stroke at baseline were followed from 1989 to 2010 for incident stroke. Overall, 297 stroke cases (179 women) were identified. Cox models with stroke-free time and risk factors recorded at baseline were used to develop stroke risk prediction models. Assessment of the developed stroke risk prediction models regarding discrimination and calibration was performed by an analogous C-statistic (C) and a version of the Hosmer-Lemeshow statistic (HL), respectively, and validated internally through use of Bootstrapping methods.

**Results:**

Age, smoking status, alcohol consumption, waist circumference, hypertension status, an-tihypertensive therapy, fasting plasma glucose, diabetes medications, high/low density lipoproteins, urinary albumin/creatinine ratio, history of coronary heart disease/heart failure, atrial fibrillation, or Left ventricular hypertrophy, and parental history of stroke were identified as the significant optimal risk factors for incident stroke.

**Discussion:**

The models produced a C = 0.761 and HL = 4.668 (p = 0.792) for women, and a C = 0.765 and HL = 9.171 (p = 0.328) for men, showing good discrimination and calibration.

**Conclusions:**

Our stroke risk prediction models provide a mechanism for stroke risk assessment designed for American Indians. The models may be also useful to other populations with high prevalence of obesity and/or diabetes for screening individuals for risk of incident stroke and designing prevention programs.

## 1. Introduction

Stroke is a major health care challenge in American Indians (AIs). Recent data indicate that AIs have a higher incidence of stroke than that of whites and blacks in the US [1]. Stroke is one of the leading causes of death as well as disability among AIs [2] [3]. Cigarette smoking, diabetes mellitus (DM), and high blood pressure are well documented modifiable risk factors for stroke [4]. We previously reported that risk factors for stroke among the AI population included age, high blood pressure, smoking, albuminuria, and diabetes [1]. Among them, DM (48.8%) and albuminuria (29.6%) were the prominent factors related to future stroke [1][ 5]as well as coronary heart disease (CHD) [6][ 7]in AIs.

A stroke prediction model utilizing routinely collected variables will assist providers who care for AIs in evaluating the risk of stroke in their patients and assist communities to design more effective and targeted interventions. Several stroke risk-assessment tools have been developed including the widely-used Framingham Risk Profile [8] [9] [10]. However, the contributions of certain common risk factors for incident stroke vary across populations [11]. Further, some risk factors/correlates have not previously been included; for example, albuminuria has been found to be significantly and independently associated with almost all of chronic diseases such as DM [12], hypertension (HTN) [13], and CHD [6] [7] in AIs. It is important to include these risk factors in the stroke prediction models for AIs.

This article presents gender-specific stroke risk prediction equations based on longitudinal data from the Strong Heart Study (SHS) during 1989–2010. A “risk calculator” from the equations will be developed for individuals to input their values of the risk factors and instantly obtain a probability (risk) of developing stroke in 10 years (will be available on the SHS Web site: http://strongheart.ouhsc.edu).

## 2. Methods

### 2.1. Study Population

The SHS is a population-based cohort study of cardiovascular disease (CVD) and its risk factors in AI tribes/communities in southwestern Oklahoma, central Arizona, and North and South Dakota. Participants (n = 3516; 2056 women) aged 45 to 74 years underwent baseline examination from 1989 to 1992. The design, inclusion and exclusion criteria of participants, survey methods, and laboratory techniques of the SHS have been described in detail [14] [15] along with methods of definition and identification of first stroke [1] [16]. Participants in the present analysis (3483; 2043 women) had no history of stroke or stroke-like events at the baseline examination. Among them, 297 (179 women) suffered an incident stroke during an average follow-up of 15.04 years (inter-quartile range 9.7 – 20.2 years) through the end of 2010. The study was approved by Institutional Review Boards of the participating institutions and tribes as well as the Indian Health Service. Informed consent was obtained from all participants.

### 2.2. Baseline Characteristics

Information on demographic factors, medical history, medication use, and personal health habits was collected by interview. A physical examination was conducted and fasting blood samples were collected for laboratory tests including lipids and lipoproteins. Anthropometric measurements were taken and sitting blood pressure (1^st^ and 5^th^ Korotkoff sounds) was measured three times consecutively using mercury sphygmomanometers (WA Baum Co) after five minutes of rest [17]. The average of the 2^nd^ and 3^rd^ systolic and diastolic blood pressure measurements were used in the analyses. HTN status was defined by the Seventh Joint National Committee on Hypertension criteria [18]: HTN if systolic blood pressure (SBP) ≥ 140 mmHg or diastolic blood pressure (DBP) ≥ 90 mmHg or on antihypertensive therapy, normal if SBP < 120 mmHg and DBP < 80 mmHg, and pre-hypertension (Pre-HTN) otherwise. DM status was defined by the American Diabetes Association diagnosis and classification guidelines [19]: DM if fasting plasma glucose (FPG) ≥ 7.0 mmol/L (126 mg/dL) or on diabetes medications, impaired fasting glucose (IFG) (or prediabetes) if 5.6 mmol/L (100 mg/dL) ≤ FPG < 7.0 mmol/L, and normal fasting plasma glucose (NFG) if FPG < 5.6 mmol/L. Micro- and macro-albuminuria were defined as urinary albumin/creatinine ratios of 30– 299 mg/g and ≥ 300 mg/g, respectively. Current smoking status was defined as smoking currently, smoking regularly, and having smoked at least 100 cigarettes in one’s entire life until the date of interview. Estimated glomerular filtration rate (eGFR) was derived based on serum creatinine that was recalibrated to an isotope dilution mass spectrometry (IDMS)-traceable serum creatinine assay [20] and using the CKD-EPI (Chronic Kidney Disease Epidemiology Collaboration) formula [21]. Participants who had CHD or congestive heart failure (HF), atrial fibrillation (AFIB), or left ventricular hypertrophy (LVH) by electrocardiography before or at the baseline examination were considered as having a history of CHD/HF, AFIB, or LVH, respectively.

### 2.3. Outcome Variables

All study participants without a prior history of stroke at the baseline examination were under follow-up surveillance for incident stroke events occurring between the date of the baseline examination and December 31, 2010. Mortality and morbidity follow-up data were available in 99.8% and 99.2% of participants, respectively.

#### 2.3.1. Fatal Stroke

Fatal events included deaths judged to be due to definite and possible stroke. Deaths occurring during the follow-up were confirmed through Indian Health Service or private hospital records and through direct contact by study personnel with participants’ families or other informants [14] [15] [22]. The process of ascertaining stroke deaths has been reported previously [1] [16] [22]. All possible stroke-related deaths were reviewed by physician members of the Strong Heart Study Mortality Review Committee and then reviewed by neurologists (D.O.W., J.P.W.) or since 2004 by a cardiologist focused on stroke (J.R.K.) for confirmation using previously described criteria [23] that differentiated eight subtypes of stroke-related events [cardioembolic infarction, subarachnoid hemorrhage, intraparenchymal hemorrhage, lacunar infarction, other unknown infarction, transient ischemic attack (TIA), unknown type of stroke, atherothrombotic infarction].

#### 2.3.2. Nonfatal Stroke

The process to confirm nonfatal stroke was similar to that for fatal stroke. Neurologists (D.O.W., J.P.W.) and later the cardiologist (J.R.K.) made up the adjudication review committee and provided the final diagnosis for non-fatal events (definite and possible non-fatal strokes) that occurred from the date of the baseline examination to Dec. 31, 2010 [14] [16] [22] [23]. Stroke event sub-types used are the same as described for fatal stroke. If more than one event happened in the same individual, the date of the earliest was considered to be the first stroke date.

### 2.4. Statistical Methods

Overall incidence rates (per 1000 persons-years) of stroke and their 95% confidence intervals and incidence rates by stroke types, gender, age groups (45 – 54, 55 – 64, and 65 – 74 years old) and centers (South/North Dakotas, Oklahoma, and Arizona) were estimated by dividing the total number of observed stroke events by the total follow-up stroke-free times (person-years) in the respective group. Stroke incidences by gender among sub-categories of each potential baseline risk factor were also estimated. Cox proportional-hazards models were used to assess univariate associations of individual risk factors with incident stroke after adjusting for age. Cox model with competing risks [24] was used in sensitivity analyses. A p-value of <0.05 was considered to be statistically significant.

### 2.5. Development of Prediction Equations

Cox proportional-hazard models were also used to assess the simultaneous association of multiple risk factors with incident stroke and to develop gender-specific stroke prediction models. Backward variable selection methods [24] with a significance level of 0.05 was used to select optimal sets of baseline risk factors for incident stroke. The potential risk factors included were, age, body mass index (BMI), waist circumference (WAIST), SBP, DBP, antihypertensive therapy (denote its indicator function as HTNRX, HTNRX = 1 if on antihypertensive therapy and = 0 if not), smoking status, physical activity, alcohol consumption, FPG, diabetes medications (denote its indicator function as DMRX, DMRX = 1 if on diabetes medications and = 0 if not), urinary albumin/creatinine ratio (UACR), eGFR, low-density lipoprotein cholesterol (LDL-C), high-density lipoprotein cholesterol (HDL-C), triglyceride (TG), history of CHD/HF, parental history of CVD, stroke, DM or HTN, history of or electrocardiogram evident atrial fibrillation (AFIB) and left ventricular hypertrophy (LVH), as well as categorization of these variables such as DM status (Yes/No; or DM, IFG and NFG), HTN status (HTN, pre-HTN, normal), and albuminuria status (macro-albuminuria, micro-albuminuria, normal). Logarithmic transformation of skewed variables was applied if needed. For the significant risk factors selected for the models, their interactions were also considered and further selected for their possible additional contributions.

### 2.6. Discrimination, Calibration, and Validation of the Prediction Equations

An analogous *C*-statistic [7] [25] was calculated to evaluate the discrimination ability of the stroke prediction models in separating those who developed stroke from those who did not. This *C*-statistic is analogous to the area under the receiver operating characteristic curve (ROC curve) based on a logistic regression. A *C*-statistic value of ≥0.7 indicates good discrimination ability, and the closer the *C* value is to 1.0, the better is the discrimination ability. A version of the Hosmer-Lemeshow χ^2^ statistic (HL-statistic) [7] [25] was computed to assess model calibration ability (or how closely the predicted probabilities reflected actual risk). Participants were divided into deciles according to their predicted probabilities of stroke in 10 years using the proposed prediction model, and the HL-statistic was calculated to compare the differences between the predicted and actual proportions of stroke events. HL-statisticvalues of <20 are considered good calibration.

In addition, the stroke prediction models were validated internally with the use of bootstrapping methods [7] [25]. Samples of the same size (n = 3483) as the original cohort were taken 1000 times from the original cohort with replacement. Then the “optimism” [7][ 25]for the *C*-statistic or the p-value for the HL-statistic was calculated based on these 1000 bootstrapping samples. A “Bootstrap-corrected statistic” then was evaluated as “the statistic from the model”-“the optimism for the statistic”. A bootstrap-corrected statistic from a model is a nearly unbiased estimate of the expected value of the statistic from the external validations of the model, with smaller “optimism” values indicating better validity of the statistic [25]. All analyses were conducted with SAS 9.4 (SAS Institute Inc., Cary, NC, USA).

## 3. Results

Table 1 shows estimated incidence rates (per 1000 person-years) of stroke for all SHS participants without prior stroke. There were no significant gender difference, but significant center differences among Arizona, Oklahoma and Dakotas with Dakotas the highest followed by Oklahoma and Arizona. Incidence rate was significantly increased with age. Incidence rates were highest for cardioembolic infarction followed by other unknown infarction, lacunar infarction and intra-parenchymal hemorrhage among identified stroke types.

Gender-specific stroke incidence rates by sub-categories of each potential baseline risk factor and its univariate association with incident stroke after adjusting for age are shown in Table 2. Age, and after adjusting for age, smoking, HTN, DM, albuminuria, history of CHD/HF, and AFIB were univariately significantly associated with incident stroke for both women and men. Alcohol consumption, HDL-C, history of LVH, and parental history of stroke were significant risk factors for women only. There were no significant univariate association of incident stroke with BMI, WAIST, physical activity, LDL-C, TG, eGFR, parental history of CVD/DM/HTN ineither women or men (data not shown).

Among these associations, after adjusting for age, for examples, those with DM had 2.25-fold higher risk than those without DM in women, and 1.65-fold higher in men; and those with macroalbuminuria or microalbuminuria had respective 3.39 or 1.66-fold higher risk than those had normal UACR in women, and 3.29 or 1.70-fold higher in men.

Gender-specific stroke prediction models are shown in Table 3. Age, current smoking, alcohol consumption, DBP and SBP as well as their interaction with HTN treatments, UACR, interaction of FPG and diabetes medications, HDL-C, history of CHD/HF, LVH and AFIB, and parental history of stroke were significantly associated with incident stroke in women. While age, WAIST, current smoking, DBP and SBP as well as their interaction with HTN treatments, Pre-HTN, UACR, diabetes medications, LDL-C, and history of CHD/HF were significantly associated with incident stroke in men.

The illustration of using the models in Table 3 to predict risk of incident stroke in 10 years for a stroke-free individual with measured risk factors or covariates was shown in Appendix.

In women, assuming the other measures in the model are the same, for examples, those with low to moderate alcohol consumption (1 - 14 drinks per week) had 50% lower risk compared with the others; and 2.5% higher risk per 10 mg/dl higher FPG among participants on diabetic medication. All terms related to blood pressures in Equation (3) (Appendix) can be rearranged as 0.02441* DBP*HTNRX + 0.00224*DBP*(1 − HTNRX) + 0.01424*SBP*(1 − HTNRX). Therefore, associations of blood pressures with incident stroke are different among those with and without antihypertensive therapy. In women, the medians UACR in the three sub-categories, normal, microalbuminuria, and macroalbu-minuria were 7, 66, and 1492 mg/g, respectively. If we use these medians as the respective reference levels of UACR in the three sub-categories, then based on the relationship between coefficient and hazard ratio in a Cox proportional-hazard model [24], the hazard ratios of macroalbuminuria vs. microalbuminuria, macroalbuminuria vs. normal, and microalbuminuria vs. normal, will be 1.174 [= Exp((Log(1492) − Log(66)) × 0.11852), where 0.11852 is the estimated coefficient for Log(UACR) in the model for women, Table 3], 1.315, and 1.120, respectively.

For men, assuming the other measures in the model are the same, the estimated hazard ratios of different levels vs. their respective reference level or hazard ratio by units change for each variable can be interpreted similarly. In addition, the age related terms in Equation (4) (Appendix) is 0.10268 × age − 0.91966 × I(age ≥ 65). Assuming the other measures in the model are the same, based on the relationship between coefficient and hazard ratio in a Cox proportional-hazard model [24], this means for every 5 years higher age stroke risk is 67% [=Exp(5 × 0.10268) − 1] higher. The association of age with incident stroke risk is dependent upon the term 0.10268*age for those aged <65, and the term 0.10268*age − 0.91966 for those aged 65 or older. Similarly, the hazard ratios of macroalbuminuria vs. microalbuminuria, macroalbuminuria vs. normal, and microalbuminuria vs. normal, based on the three medians (5, 70, and 873 mg/g for normal, microalbuminuria, and macroalbuminuria sub-categories in men, respectively) are 1.159, 1.354, and 1.168, respectively.

The C-statistics from the models for women and men are 0.761 and 0.765, respectively, indicating good discrimination ability. The respective HL-statistics 4.668 (p = 0.792) and 9.171 (p = 0.328) show good calibration ability of the models.

**Figure 1:**
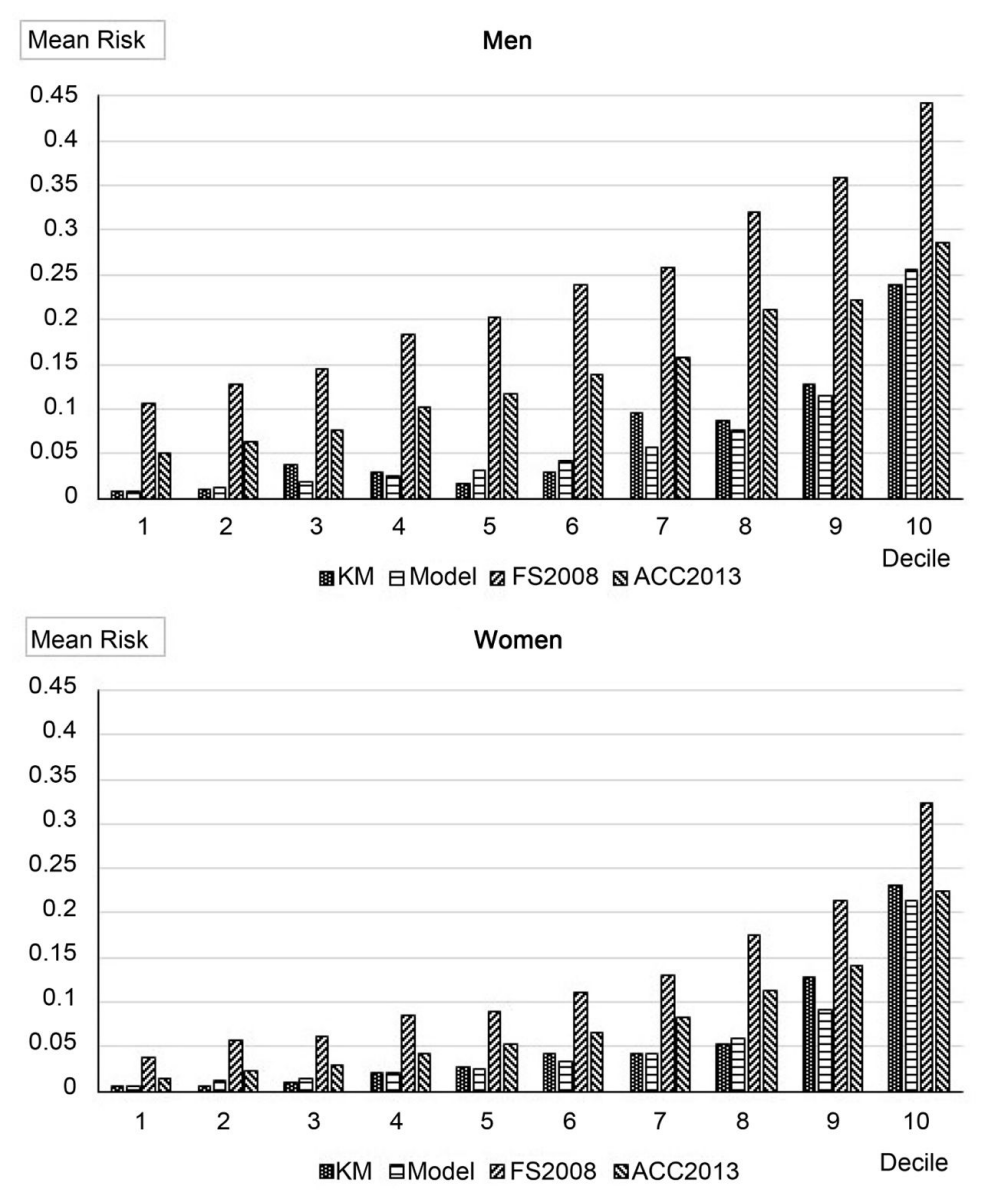
Calibration by deciles of model-based predicted probabilities of stroke event in 10 years. “KM” denotes observed risk (by using Kaplan-Meier method). “Model” denotes the models in Table 3 based predicted, “FS2008” the Framingham 2008 models based predicted, and “ACC2013” the ACC/AHA 2013 models (for White) based predicted de-cile specific risk means in deciles.

Figure 1 displays the calibration plots comparing actual observed risk and predicted decile specific means of risk in men and women. The internal validation results based on the bootstrapping method show a bootstrap-corrected C-statistic of 0.7456 (after subtraction of optimism of 0.01489) for women, and 0.7458 (after subtraction of optimism of 0.01949) for men. The respective bootstrap-corrected p-value for HL-statistic was 0.9998 (optimism = −0.2075) for women and 0.4768 (optimism = −0.14875) for men. These C-statistics and p-values for HL-statistic with their bootstrap-corrections, and the small optimism values indicate good calibration and discrimination ability as well as stability of the prediction models.

We also applied Framingham 2008 [9] or American College of Cardiology (ACC)/American Heart Association (AHA) 2013 [10] prediction models (with published estimated coefficients for risk factors and values of baseline function at *t* = 10) to predict stroke risk in AIs. The applications of Framingham 2008 prediction models produced a C-statistic= 0.701 and a HL-statistic= 109.73 (p< 0.0001) for women, and C = 0.706 and HL-statistic = 281.9 (p < 0.0001) for men; and those applications of ACC/AHA 2013 prediction models for White produced a C-statistic=0.705 and a HL-statistic = 29.82 (p < 0.00023) for women, and C= 0.709 and HL-statistic= 82.3 (p< 0.0001) for men, while those for Black produced a C-statistic = 0.705 and a HL-statistic = 91.2 (p < 0.0001) for women, and C = 0.711 and HL-statistic = 80.6 (p < 0.0001) for men. The predicted decile specific means of risk in men and women from Framingham 2008 or ACC/AHA 2013 (for White) models are also showed in Figure 1.

To explore performance of the generated models in predicting risk of non-hemorrhagic incident strokes only, a sensitivity analyses was conducted by treating incident hemorrhagic stroke as a competing risk (and hence as censored event competing with non-hemorrhagic incident stroke) [24]. The generated models produced a C = 0.763 and a HL-statistic = 4.877 (p = 0.7706) for women, and C = 0.771 and HL-statistic = 5.558 (p = 0.6966) for men, and therefore there were better discrimination and calibration scores for the generated models for non-hemorrhagic incident strokes compared to those respective Cs and HLs for all incident strokes shown in Table 3.

## 4. Discussion

The new prediction models for incident stroke based on data routinely acquired in a clinical setting should prove to be helpful for care providers to evaluate stroke risk of their patients. Of perhaps equal importance, they will allow providers to further reinforce preventive measures such as smoking cessation, preventing or managing diabetes, and controlling blood pressure and LDL levels.

Some of these risk factors such as age, smoking status, SBP, DBP, HTN status, DM status, history of CHD/HF, AFIB, and LVH have also been reported as stroke risk factors [1] [8] [10]; and LDL-C, alcohol consumption and albuminuria in other studies [1] [5] [26] [27] [28]. Among them, albuminuria is especially and significantly associated with incident stroke in AIs. We found that SHS participants who had macroalbuminuria or microalbuminuria had respectively 3.39 or 1.66 times higher risk of incident stroke than those with normal UACR in women, and 3.29 or 1.70 times in men from the age-adjusted univariate analyses (Table 2). These hazard ratios remained to be 1.315 and 1.120 in women and 1.354 and 1.168 times in men after adjusting for the other risk factors in the models (Table 3) as explained in Results section. The hazard ratios of macroalbuminuria vs. normal UACR were almost equal to those of AFIB vs. not AFIB. Given that AFIB constitutes a previously well-known significant and crucial risk factor for incident stroke [8], the considerable association of albuminuria to stroke in this population cannot be ignored. The significant terms of diabetes medications in men and the interaction of FPG with diabetes medications in women remained in the final models. Which show DM is significantly associated with incident stroke risk and suggest that controlling FPG, especially in those with DM and on diabetes medications, is very important in preventing incident stroke. Our models identified significant independent contributions and combined effects of these risk factors in predicting risk of incident stroke after adjusting for the other risk factors in the respective models. Our models were also somewhat better at predicting non-hemorrhagic strokes than total strokes. This is likely because the majority of stroke cases were non-hemorrhagic strokes.

There are some interesting gender differences from this study. Table 2 and Table 3 show that low-moderate alcohol consumption (1 – 14 drinks for female) may be protective against incident stroke in women only. The beneficial effect of low-moderate alcohol consumption in women is consistent with previous findings, but the lack of a significant association for men contradicts those reported in the literature [27]. HDL-C was associated univariatly and multivariately with incident stroke only in women while LDL-C was associated only in men. The reasons for these gender differences are unclear and require further investigation.

Our models had improved predictive value compared to either the Framing-ham 2008 [9] or ACC/AHA 2013 [10] models when examined in AI. The lower performance of the Framingham or ACC/AHA models may be affected by their miscalibration [29] (that is, the average predicted risk from these models are not close to the stroke event rate in AI). The 10 years stroke event rate in AI were 0.043 for women and 0.050 for men, while the average predicted risk from the Framingham models were 0.128 and 0.139, and from ACC/AHA models (for White) 0.078 and 0.142, respectively. The miscalibration can also be seen in Figure 1 and from their large HL-statistics and respective significant p-values mentioned in Results section.

We did not use a reclassification statistic such as net reclassification improvement (NRI) [30] to compare our models with those reported in the literature such as the Framingham or ACC/AHA models. The reasons are due to those reported issues related to miscalibration on clinical use of a risk equation (as we discussed above) in different populations, comparing different models such as different outcomes or population groups used in reported models, and uncertainty about how to draw proper 10-year stroke risk cutoff points [29]. In addition, the NRI is the difference of Youden indexes from two models for a binary classification with a cutoff probability. The problems associated with NRI include concerns about statistical invalidity in real and simulated data, inadequately accounting for clinically important differences in shifts among risk categories if there are three or more risk categories, and other controversies [29] [30][ 31][ 32].

## 5. Conclusion

Our generated stroke prediction models based on the data from the SHS provide a stroke risk appraisal specific for a population with high prevalence of obesity, diabetes, and renal disease. With the increasing of incidence and prevalence of obesity and diabetes in the US, we believe that our generated prediction models would provide an additional helpful assessment tool for other similar populations. Although our generated stroke prediction models are internally validated, they should be tested and validated in other populations.

## Figures and Tables

**Table 1:** Incidence rate of stroke (per 1000 person-years) during averaged 15.04 years of follow-up for SHS stroke-free baseline participants aged 45 – 74 years.

Group	N	No. of Stroke Events	Total Follow-up Person-Years	Incidence Rate	95% Confidence Interval	
**Stroke event subtype**						
**Ischemic stroke**						
Atherothrombotic infarction	3483	13	53,910.94	0.24	0.11	0.37
Cardioembolic infarction	3483	107	53,335.39	2.01	1.63	2.39
Lacunar infarction	3483	42	53,638.54	0.78	0.54	1.02
Other unknown infarction	3483	43	53,768.89	0.80	0.56	1.04
**Hemorrhagic stroke**						
Intraparenchymal hemorrhage	3483	29	53,891.19	0.54	0.34	0.74
Subarachnoid hemorrhage	3483	5	53,934.42	0.09	0.01	0.17
**Unknown type of stroke**	3483	57	53,778.16	1.06	0.78	1.34
**Transient ischemic attack (TIA)**	3483	1	53,976.47	0.02	−0.02	0.06
**All Stroke**	3483	297	52,398.72	5.67	5.03	6.31
**Women**	2043	179	31,877.86	5.62	4.80	6.44
**Men**	1440	118	20,520.86	5.75	4.71	6.79
**Age Group**						
45 – 54	1701	99	28,205.97	3.51	2.82	4.20
55 – 64	1155	124	16,749.30	7.40	6.10	8.70
65 – 74	627	74	7443.45	9.94	7.67	12.21
**Center**						
South/North Dakotas	1509	150	21,876.84	6.86	5.76	7.96
Oklahoma	1508	125	23,700.31	5.27	4.35	6.19
Arizona	466	22	6821.57	3.23	1.88	4.58

**Table 2:** Incidence rates (per 1000 person-years) of stroke by gender and sub-categories of potential baseline risk factors, and their age-adjusted individual association to incident stroke.

		Women					Men			
Variable	Incidence Rate	95% Confidence Interval		Hazard Ratio*	p*	Incidence Rate	95% Confidence Interval		Hazard Ratio*	p*
**Age: 45** – **54**	3.7	2.77	4.63	Ref.		3.25	2.22	4.28	Ref.	
55 – 64	6.65	5.09	8.21	1.824	**0.0006**	8.67	6.36	10.98	2.656	**<0.0001**
65 – 74	9.89	7.09	12.69	2.784	**<0.0001**	10.05	6.19	13.91	3.088	**<0.0001**
**Current Smoking:** No	4.71	3.78	5.64	Ref.		4.82	3.57	6.07	Ref.	
Yes	7.54	5.88	9.2	1.895	**<0.0001**	6.96	5.17	8.75	1.689	**0.0056**
**Alcohol (drinks/per week)**										
None	6.84	5.71	7.97	Ref.		6.88	5.17	8.59	Ref.	
1 – 14 Female/1–21 Male	3.17	2.05	4.29	0.567	**0.0051**	5.1	3.66	6.54	0.901	**0.5966**
>14 Female/>21 Male	6.46	0.13	12.79	1.428	**0.4882**	3.86	0.47	7.25	0.721	**0.4853**
**HTN:** Normal	3.49	2.42	4.56	Ref.		2.53	1.16	3.9	Ref.	
Pre-HTN	5.32	3.84	6.8	1.383	**0.1260**	6.37	4.6	8.14	2.419	**0.0046**
HTN	8.26	6.53	9.99	1.891	**0.0012**	7.26	5.32	9.2	2.407	**0.0047**
**DM:** No	3.86	2.99	4.73	Ref.		4.63	3.5	5.76	Ref.	
Yes	9.11	7.34	10.88	2.247	**<0.0001**	8.19	5.96	10.42	1.648	**0.0077**
**Albuminuria:**										
Normal	4.5	3.67	5.33	Ref.		4.72	3.67	5.77	Ref.	
Micro	7.94	5.38	10.5	1.657	**0.0081**	8.64	5.11	12.17	1.702	**0.0263**
Macro	14.43	8.66	20.2	3.388	**<0.0001**	15.2	7.24	23.16	3.288	**<0.0001**
**HDL-C:**										
≥1.554 (60 mg/dl)	3.42	1.92	4.92	Ref.		7.3	3.33	11.27	Ref.	
1.036 – 1.528 (40 – 59 mg/dl)	5.13	4.08	6.18	1.577	**0.0653**	5.36	3.85	6.87	0.763	**0.3868**
<1.036	8.08	6.08	10.08	2.592	**0.0002**	5.74	4.22	7.26	0.821	**0.5226**
**History of CHD/HF: No**	5.22	4.41	6.03	Ref.		5.05	4.04	6.06	Ref.	
Yes	16.98	9.14	24.82	3.009	**<0.0001**	15.81	9.05	22.57	2.407	**0.0003**
**History of AFIB: No**	5.54	4.72	6.36	Ref.		5.63	4.6	6.66	Ref.	
Yes	28.54	−3.75	60.83	4.717	**0.0079**	33.13	4.36	70.62	3.390	**0.0385**
**History of LVH:** No	5.32	4.51	6.13	Ref.		5.7	4.66	6.74	Ref.	
Yes	37.68	15.41	59.95	5.916	**<0.0001**	12.67	4.88	30.22	1.475	**0.5872**
**Parental History of Stroke**										
No	5	4.1	5.9	Ref.		5.61	4.46	6.76	Ref.	
Yes	7.36	5.51	9.21	1.466	**0.0153**	6.28	3.91	8.65	1.086	**0.7068**

AFIB, atrial fibrillation; Albuminuria, normal if urinary albumin-to-creatinine ratio (UACR) < 30 mg/g, microalbuminuria if 30 ≤ UACR < 300, and macroalbuminuria if UACR ≥ 300; CHD, coronary heart disease; DBP, diastolic blood pressure; DM, diabetes, DM status, DM if fasting plasma glucose (FPG) ≥ 7.0 mmol/L (126 mg/dL) or on diabetes medications, impaired fasting glucose (IFG) (or prediabetes) if 5.6 mmol/L (100 mg/dL) ≤ FPG < 7.0, and normal fasting plasma glucose (NFG) if FPG < 5.6; HDL-C, high-density lipoprotein cholesterol; HF, heart failure; HTN, hypertension, HTN status, HTN if systolic blood pressure (SBP) ≥ 140 mmHg or DBP ≥ 90 or on antihypertensive therapy, normal if SBP < 120 and DBP < 80, and prehypertension (Pre-HTN) otherwise; LVH, left ventricular hypertrophy; P, P-value; Ref., reference level. *Hazard Ratios and *P*-values are from the Cox models for assessing age-adjusted association of incident stroke with the individual risk factors. The p-values are from testing whether a hazards ratio of the respective level vs. its reference level is significantly different from one (e.g. P = 0.0012 for the hazards ratio of 1.891 for HTN vs. Normal (reference level) in women).

**Table 3:** Cox proportional hazards models for stroke-free time.

Parameter	Estimated Coeff.	SE	p	Hazard Ratio*	95% Confidence Interval	
	**Women, S_0_(10) = 0.9996979618**					

Age (year)	0.05278	0.0113	<0.0001	1.302	1.165	1.455
Current smoking	0.98181	0.1659	<0.0001	2.669	1.926	3.693
Alcohol consumption 1 – 14 drinks/per week	−0.69887	0.2151	0.0012	0.497	0.320	0.746
DBP (mmHg)	0.02441	0.0080	0.0022	1.025	1.009	1.041
DBP*(1 − HTNRX) (mmHg)	−0.02217	0.0106	0.0373	0.978	0.958	0.999
SBP*(1 − HTNRX) (mmHg)	0.01424	0.0060	0.0176	1.014	1.002	1.026
Log(UACR) (mg/g)	0.11852	0.0457	0.0094	1.126	1.029	1.231
FPG* DMRX (mg/dl)	0.00243	0.0007	0.0008	1.025	1.010	1.039
HDL-C (mg/dl)	−0.02063	0.0072	0.0043	0.814	0.706	0.937
History of CHD/HF	0.81251	0.2963	0.0061	2.254	1.202	3.875
History of LVH	1.15170	0.4357	0.0082	3.164	1.207	6.862
History of AFIB	1.60921	0.5964	0.0070	4.999	1.212	13.650
Parental History of Stroke	0.37490	0.1653	0.0234	1.455	1.046	2.002
**C-statistic**	0.761					
**Hosmer-Lemeshow statistic**	4.668	p = 0.792				

	**Men, S_0_(10) = 0.9999426319**					

Age (year)	0.10268	0.0189	<0.0001	1.671	1.388	2.011
Age ≥ 65	−0.91966	0.3349	0.0060	0.399	0.205	0.766
WAIST (cm)	−0.01881	0.0088	0.0322	0.981	0.964	0.998
Current smoking	0.65459	0.2011	0.0011	1.924	1.296	2.857
DBP* HTNRX (mmHg)	0.02661	0.0098	0.0064	1.027	1.007	1.047
SBP*(1 − HTNRX) (mmHg)	0.01566	0.0061	0.0100	1.016	1.004	1.028
Pre-HTN	0.53292	0.2272	0.0190	1.704	1.096	2.678
Log(UACR) (mg/g)	0.13446	0.0533	0.0117	1.144	1.029	1.268
Diabetes Medications	0.79694	0.2402	0.0009	2.219	1.374	3.529
100 ≤ LDL-C < 130 mg/dl	−0.49104	0.2123	0.0207	0.612	0.398	0.918
History of CHD/HF	0.98687	0.2648	0.0002	2.683	1.558	4.420
**C-statistic**	0.765					
**Hosmer-Lemeshow statistic**	9.171	p = 0.328				

AFIB, atrial fibrillation; CHD, coronary heart disease; Coeff, coefficient; DBP (SBP), diastolic (systolic) blood pressure; DBP*HTNRX, DBP*(1-HTNRX), SBP*(1-HTNRX), the interaction of DBP/SBP and anti-hypertensive therapy, where HTNRX=1 if on antihypertensive therapy and = 0 if not; FPG, Fasting plasma glucose; FPG*DMRX, the interaction of FPG and diabetes medications, where DMRX = 1 if on diabetes medications and = 0 if not; HDL-C, high-density lipoprotein cholesterol; HF, heart failure; HTN, hypertension; HTN status, HTN if SBP ≥ 140 mmHg or DBP ≥ 90 or on antihypertensive therapy, normal if SBP < 120 and DBP < 80, and prehypertension (Pre-HTN) otherwise; LDL-C, low-density lipoprotein cholesterol; LVH, Left ventricular hypertrophy; P, p-value; *S*_0_(10), the baseline stroke-free time function evaluated at *t* = 10 years; SE, standard error; UACR, urinary albumin creatinine ratio; WAIST, waist circumference.

* The unit used to calculate Hazard Ratio is 5 years for age, and 10 mg/dl for FPG and HDL-C.

## Appendix

The stroke-free time distribution function based on the Cox proportional-hazard model from the final selected model was as follows [24]

(1) $$S(t,x)={\left[S_{0}(t)\right]}^{\exp\left(\sum_{i=1}^{p}b_{i}x_{i}\right)}$$

where *S*_0_(*t*) is the estimated baseline stroke-free time function, ***x*** = (*x*_1_, *x*_2_, ⋯, *x_p_*) are the final selected optimal set of risk factors, and *b*_1_, *b*_2_, ⋯, *b_p_* are their respective estimated coefficients. *S*_0_(*t*) was estimated according to a method proposed by Breslow [24].

Based on Equation (1), the probability that an individual will develop stroke in *t*years is estimated by the following equation

(2) P(t,x)=1-S(t,x)

with the given set of risk factors ***x*** of the individual.

From Table 3 the summation terms in Equation (1) for women and men are:

Women:

(3) $$\begin{array}{l} \sum b_{i}x_{i}=0.05278*\text{Age}+0.98181*I(\text{Current}\;\text{smoking})\\ -0.69887*I(\text{Alcohol}\;\text{consumption}\;1-14\;\text{drinks}/\text{per}\;\text{week})\\ +0.02441*\text{DBP}-0.02217*\text{DBP}*(1-\text{HTNRX})\\ +0.01424*\text{SBP}*(1-\text{HTNRX})+0.11852*\text{Log}(\text{UACR})\\ +0.00243*\text{FPG}*\text{DMRX}-0.02063*\text{HDL-C}\\ +0.81251*I(\text{History}\;\text{of}\;\text{CHD}/\text{HF})+1.15170*I(\text{LVH})\\ +1.60921*I(\text{AFIB})+0.37490*I(\text{parental}\;\text{history}\;\text{of}\;\text{stroke}), \end{array}$$

Men:

(4) $$\begin{array}{l} \sum b_{i}x_{i}=0.10268*\text{Age}-0.91966*I(\text{Age}\geq 65)-0.01881*\text{WAIST}\\ +0.65459*I(\text{Current}\;\text{smoking})+0.02661*\text{DBP}*\text{HTNRX}\\ +0.01566*\text{SBP}*(1-\text{HTNRX})+0.53292*I(\text{Pre-HTN})\\ +0.13446*\text{Log}(\text{UACR})+0.79694*\text{DMRX}\\ -0.49104*I(100\leq \text{LDL-C}<130\;\text{mg}/\text{dl})\\ +0.98687*I(\text{History}\;\text{of}\;\text{CHD}/\text{HF}), \end{array}$$

where I(.) is the indicator function, which equals 1 if the condition in the parentheses is met and 0 otherwise.

To illustrate the use of models in Table 3 and Equations (1) to (4) to predict risk of incident stroke in 10 years for, say, a stroke-free man who is 60.5 years old smoker, and has waist circumference = 100 cm, SBP/DBP = 183/114 mmHg and take hypertension medications, not using DM medications, UACR = 160.6 mg/g, LDL-C = 162 mg/dl, and the history of CHD/HF, by applying Equation (4), the summation term in Equation (1) for this man equals 0.10268 × 60.5 − 0.91966 × 0 − 0.01881 × 100 + 0.65459 × 1 + 0.02661 × 114 × 1 + 0.01566 × 183 × (1 − 1) + 0.53292 × 0 +0.13446 × Log(160.6) + 0.79694 × 0 − 0.49104 × 0 + 0.98687 × 1 = 9.687187. From Equations (2) and (1), his probability (risk) of developing stroke in 10 years will equal to

$$\begin{array}{l} P(\text{developing}\;\text{stroke}\;\text{in}\;10\;\text{years},x)=1-S(10,x)=1-S_{0}{\left(10\right)}^{\exp(9.687187)}\\ =1-0.999942632^{\exp(9.687187)}=0.603158, \end{array}$$

where *S*_0_(10) (=0.999942632, Table 3) is the baseline stroke-free time function evaluated at *t*=10 for the model. The predicted probability of 60.3% is about 5 times the average probability 12.7% (Table A1) risk of developing incident stroke in 10 years for a man this age. This calculation can be easily conducted by a MS Excel work sheet or directly using stroke risk calculator that will be created on the SHS web site.

**Table A1:** Average predicted probability (risk) of developing incident stroke in 10 years.

Age	Women	Men
45 – 49	0.0224	0.0240
50 – 54	0.0350	0.0396
55 – 59	0.0507	0.0745
60 – 64	0.0714	0.1268
65 – 69	0.0860	0.0887
70 – 74	0.1202	0.1380
